# “We want our freedom back, that’s our only need”: a qualitative study of health and social needs among asylum seekers and undocumented migrants crossing the borders from Belarus to Lithuania

**DOI:** 10.3389/fpubh.2024.1371119

**Published:** 2024-05-02

**Authors:** Rabie Adel El Arab, Rita Urbanavice, Agne Jakavonyte-Akstiniene, Marija Skvarcevskaja, Donatas Austys, Erica Briones-Vozmediano, Esther Rubinat-Arnaldo, Natalja Istomina

**Affiliations:** ^1^Faculty of Nursing and Physiotherapy, University of Lleida, Lleida, Spain; ^2^Healthcare Research Group (GRECS), Institute for Biomedical Research (IRBLleida), Lleida, Spain; ^3^Health and Social Services for Asylum Seekers Research Group, Vilnius University, Vilnius, Lithuania; ^4^Department of Health Management and Informatics, AlMoosa College of Health Sciences, Al Ahsa, Saudi Arabia; ^5^Institute of Health Sciences, Faculty of Medicine, Vilnius University, Vilnius, Lithuania

**Keywords:** asylum seekers, Lithuania, undocumented migrant, COVID-19, health needs, social needs

## Abstract

**Background:**

The influx of undocumented migrants and asylum seekers into Lithuania, particularly during the COVID-19 pandemic, presents unique public health challenges. This study employs the Social Determinants of Health framework to explore the healthcare and social needs of this vulnerable population.

**Methods:**

In May 2022, we carried out a qualitative study through semi-structured interviews with asylum seekers across four centers in Lithuania. Employing both purposive and snowball sampling techniques, we selected participants for our investigation. The study comprised 21 interviews—19 conducted in Arabic and 2 in English—with durations ranging between 20 and 40 min each. We audio-recorded all interviews, transcribed them verbatim, and subsequently performed a thematic analysis using Atlas.ti software. This process of design and analysis strictly followed the principles of thematic analysis as outlined by Braun and Clarke, guaranteeing methodological precision and rigor.

**Findings:**

21 interviews revealed critical insights into the healthcare access challenges, mental health issues, and social integration barriers faced by the participants. Key themes included ‘Healthcare Needs and the Impact of the COVID-19 Pandemic ‘and ‘Social needs and Aspirations Amidst Pandemic-Induced Uncertainty ‘. The findings highlight the multifaceted healthcare and social needs of asylum seekers, juxtaposed against significant barriers they face. Access to medical services is hindered by long waiting times and financial constraints, especially for specialized care such as dental services. Communication issues during medical appointments due to language barriers and the lack of gender-specific healthcare, such as access to gynecological services, further exacerbate the challenges. Additionally, the COVID-19 pandemic introduces hurdles such as limited testing, isolation measures, language-specific information barriers, and insufficient social distancing practices. Mental health has emerged as a critical concern, with asylum seekers reporting significant stress and emotional exhaustion due to uncertainty and restrictive living conditions. Social needs extend to delayed asylum application processes, inconsistent language education opportunities, inadequate clothing, and nutrition that lacks cultural sensitivity, and living conditions characterized by overcrowding and insufficient facilities. The restricted freedom of movement within asylum seeking centres severely impacts their psychological well-being, underscoring a deep longing for autonomy and a better life despite the myriad of challenges faced.

**Discussion:**

The study illustrates the complex interplay between migration, health, and social factors in the context of a global pandemic. It highlights the need for culturally sensitive healthcare services, mental health support, and structured language education programs. Offering educational avenues alongside language courses for children and adults is essential for fostering social inclusion and securing economic prosperity. Addressing the challenge of language barriers is of utmost importance, as these barriers significantly impede undocumented migrants’ and asylum seekers employment opportunities and their access to crucial services. The findings emphasized immigration as a health determinant and underscored the importance of inclusive health policies and advocacy for undocumented migrants and asylum seekers’ rights and needs.

**Conclusion:**

There is an urgent need for comprehensive policies and practices that are grounded in the principles of equity, compassion, and human rights. Additionally, advocating for practice adaptations that are culturally sensitive, linguistically inclusive, and responsive to the unique challenges faced by undocumented migrants and asylum seekers. As global migration continues to rise, these findings are crucial for informing public health strategies and social services that cater to the diverse needs of this vulnerable population.

## Introduction

Undocumented migrants and asylum seekers, constituting a vulnerable population group, encounter formidable barriers to accessing healthcare, owing to their legal status, language impediments, and limited resources (1). The health needs of these individuals frequently go unaddressed, leading to adverse outcomes and heightened risks of morbidity and mortality (1–4). Legal constraints significantly impede the health and social integration of these individuals, with restrictive immigration policies potentially dissuading them from seeking essential healthcare, thereby posing notable public health risks (5–7). This predicament not only contributes to tangible health issues but also fosters profound feelings of isolation, marginalization, and an overarching sense of insecurity among undocumented migrants and asylum seekers (Supplementary Appendix 1) (8).

Undocumented migrants and asylum seekers grapple with a myriad of social determinants of health, including housing instability, limited employment opportunities, and educational barriers. These factors create a cycle of poverty and social exclusion, profoundly influencing overall well-being (9) This cycle of adversity significantly heightens the vulnerability of undocumented migrants and asylum seekers to a spectrum of health challenges, spanning both chronic and infectious diseases. Specific diseases, such as cardiovascular diseases, diabetes, tuberculosis, HIV/AIDS, hepatitis B and C, malaria, mental disorders, substance abuse, and sexual and reproductive health problems are prevalent among this population (10–12).

The experiences associated with asylum seeking can profoundly influence an individual’s mental health. The diverse challenges faced by those navigating displacement, including instances of violence, persecution, and forced separation from family, are recognized as potential triggers for mental health issues such as post-traumatic stress disorder (PTSD), anxiety, and depression (13, 14).

Navigating an unfamiliar environment can intensify the myriad challenges faced by undocumented migrants and asylum seekers. These challenges include discrimination, social isolation, and economic hardship, which collectively contribute to heightened mental distress among this vulnerable population. For instance, individuals may encounter prejudiced attitudes, experience exclusion from social circles, and grapple with financial struggles, all of which significantly impact their psychological well-being (15, 16). Moreover, the fear of deportation and the constant uncertainty surrounding their legal status further intensify mental health concerns. This perpetual state of apprehension can lead to social anxiety and hypervigilance among undocumented migrants and asylum seekers, affecting their overall mental and emotional resilience (15, 16). The COVID-19 pandemic has further strained already limited health resources, making it even more challenging for these populations to receive healthcare (1). Globally, 15.9 million people (14.7–17.2 million) died from all causes related to the COVID-19 pandemic during 2020 and 2021. This number includes deaths directly attributable to SARS-CoV-2 infection and those indirectly related to other social, economic, or behavioral changes associated with the pandemic (17).

The right to health is universally recognized and applies to everyone, irrespective of legal status or nationality. International law, aligning with the 2030 Agenda for Sustainable Development (18), ensures universal access to healthcare. Nevertheless, the provision of health care for undocumented migrants and asylum seekers varies considerably among European Union (EU) countries (19). To illustrate, some countries may have more inclusive policies, while others might have restrictive practices.

Culturally sensitive health services play a pivotal role in addressing these disparities and fostering resilience (20). Ensuring that healthcare is attuned to the cultural backgrounds of undocumented migrants and asylum seekers is crucial for overcoming challenges related to accessibility and effectiveness. This may involve linguistic considerations, understanding diverse health beliefs, and tailoring services to meet the unique needs of this population.

## Background

The surge of undocumented migrants and asylum seekers from Belarus into Latvia, Lithuania, and Poland has been unfolding since mid-2021 and has brought to the forefront the pressing issue of their health and well-being (21). The following characteristics have been observed among the undocumented migrants who arrived in Lithuania from June 2021 to December 2023 (22). There were 4,614 undocumented migrants, the majority of whom originated from Middle Eastern countries, specifically Iraq and Syria. Iraq topped the list of countries of origin for migrants, with a total of 2,864 persons, followed by 193 from Syria. Analysis of the demographic data reveals a gender disparity among undocumented migrants: 72% were males, 28% were females. Additionally, 90% of them have requested asylum (23). Thus, Understanding the unique health needs of both genders is crucial for providing effective care and support. In 2021, the Lithuanian government established five centers for asylum seekers who have crossed the Belarussian border into Lithuania (24). Three of those centers were under the control of the Ministry of the Interior of the Republic of Lithuania. The other two centers were managed by the Ministry of Social Security and Labor (24).

Foreign nationals who are permanent residents in Lithuania and/or are insured by Lithuania’s compulsory health insurance plan, including those seeking asylum or refugees, are able to access vaccination services, such as COVID-19 vaccinations, in Lithuania. This service is also available to all insured citizens of other EU member states (25).

The surge of undocumented migrants and asylum seekers crossing from Belarus into Lithuania introduced a host of challenges, particularly concerning the health and well-being of these individuals. To effectively address their immediate healthcare needs and long-term mental health, a precise and targeted set of measures must be implemented.

It is imperative to recognize that the exclusion of undocumented migrants and asylum seekers or the limited inclusion in healthcare and social systems may exert additional pressure on health and social systems. Additionally, addressing structural factors, including but not limited to living conditions, is crucial. Exploring the specific ways in which these factors influence health outcomes and proposing targeted interventions can contribute to a more effective response.

## Conceptual framework

The conceptual framework of this study centers on the Social Determinants of Health (SDH), which delineate the conditions shaping an individual’s life from birth to aging. These conditions are intricately entwined with the global, national, and local distribution of financial resources, power, and other assets (26). The prospect exists that SDH forms the crux of health inequities (27). This investigation adopts the Commission on Social Determinants of Health (CSDH) framework (28), a model pioneered by the World Health Organization. Envisioned as a tool for comprehending how political and socioeconomic policies impact the health and social requisites of migrants, the CSDH framework facilitates the nuanced analysis and communication of the intricate phenomenon of social determinants of health (28).

Within the CSDH framework, determinants are classified into two categories: structural and intermediary. Structural health determinants are influenced by cultural and social values, public policies, and political systems, all of which shape a spectrum of “structural mechanisms” including income, education, and occupation (28). These mechanisms perpetuate social stratification, fortify power dynamics, and sustain societal privileges, reproducing inter-group interactions. Gender discrimination is a pervasive element in numerous societies, subjecting women and girls to systematic disparities in power, prestige, and resource allocation. The repercussions of discrimination are profound, manifesting in immediate and severe health consequences such as rape, sexual exploitation, forced marriages, and gender-based domestic violence. Similarly, belonging to marginalized racial or ethnic groups profoundly impacts an individual’s social status, economic prospects, and life trajectory in societies characterized by racial discrimination and exclusion. Health outcomes for oppressed racial and ethnic groups tend to be markedly lower than those for more privileged groups or the general population. Health inequity is contingent upon sociopolitical context and structural mechanisms. Furthermore, these determinants interconnect with another set of determinants known as intermediary determinants of health (28).

These intermediary factors encompass environmental aspects like neighborhood characteristics, housing quality, and work environment; socio-psychological factors such as psychosocial stressors and stressful living conditions; behavioral factors including nutrition, physical activity, smoking, and alcohol consumption; genetic and aging factors; and factors related to the healthcare system, including accessibility and affordability of services. As defined in the framework, intermediary determinants are social factors influencing health and contributing to unequal exposure and vulnerability to harmful conditions such as lack of access to safe drinking water, poor dietary choices, and unhealthy living conditions (28).

Importantly, immigration is positioned as a determinant of health in its own right for the purpose of achieving substantial enhancements in health outcomes (29). The immigrant experience significantly shapes behavior and modifies the impact of other social positions, such as socioeconomic status, given the ambiguous relationship between immigrants and state institutions, including healthcare (29). This holistic framework informs the study’s exploration of the intricate dynamics influencing the health and well-being of undocumented migrants and asylum seekers.

### Aim

The aim of this study was to thoroughly investigate the healthcare and social needs of undocumented migrants and asylum seekers who had crossed the borders into Lithuania from Belarus.

### Objectives

To explore undocumented migrants and asylum seekers’ healthcare and social needs in Lithuania.

To explore undocumented migrants and asylum seekers’ perceptions and experiences regarding the impact of the COVID-19 pandemic on their health and social well-being.

## Methods

### Study design

The study employed a qualitative approach, semi-structured interviews to comprehensively explore the healthcare and social needs of undocumented migrants and asylum seekers in Lithuania. The qualitative approach enables an in-depth exploration of undocumented migrants and asylum seekers’ lived experiences, offering nuanced insights into their healthcare and social needs. This methodology excels at uncovering complex dynamics and personal narratives, which quantitative methods may fail to capture, thereby offering a comprehensive understanding of the participants’ challenges and needs (30). The research team meticulously developed the semi-structured interview guide in English, informed by existing literature and internal discussions. To ensure linguistic accuracy, the guide was translated into Arabic and back translated by two bilingual speakers. The interview guide covered a diverse range of topics, including healthcare access, existing healthcare-related needs, communication challenges, diet and meals concerns, social activities, social welfare, COVID-19 information, and obstacles to integration in Lithuania. The selected participants, undocumented migrants, and asylum seekers in Lithuania, were most appropriate because they directly experience the phenomena under investigation. Their firsthand accounts and perspectives provide critical insights into the healthcare access challenges, mental health issues, and social integration barriers they face, especially during the COVID-19 pandemic.

### Sampling methods

Purposive sampling and a snowball sampling approach, as per Bryman and Bell (30), were employed for participant selection. In the recruitment process for our study, we employed a two-pronged strategy combining purposive and snowball sampling techniques to ensure a diverse and representative sample of undocumented migrants and asylum seekers. Initially, purposive sampling was utilized to select participants who met specific criteria relevant to our study.

Following the initial recruitment, we implemented snowball sampling to leverage the networks of our purposively selected participants. Participants were asked to recommend others within their community who met our study criteria and might be willing to share their experiences. This technique was particularly valuable in reaching a broader segment of the undocumented migrant and asylum seeker population.

The combination of these sampling methods enabled us to construct a robust sample that not only met our predefined criteria but also captured a wider range of experiences and perspectives among the study population. This approach enhanced the depth and breadth of our qualitative analysis, contributing to a comprehensive understanding of the complex issues faced by undocumented migrants and asylum seekers.

### Data collection

Permission was obtained from the management of asylum seeker centers, and two members of the research team (RA and RU) visited the centers to invite participants. Information sheets were distributed, and the research team explained the study to potential participants. The study was conducted in May 2022, ensuring a comprehensive exploration of the healthcare and social needs of undocumented migrants and asylum seekers in Lithuania. The participants had the option to conduct the interviews either in Arabic or in English, interviews were between 20 and 40 min and were audio-recorded for verbatim transcription. Language proficiency was assessed informally through initial communications with potential participants. English and Arabic were chosen based on the predominant languages spoken by the asylum seeker population in the study setting, ensuring effective communication and understanding during the interviews. Additionally, the interviewer (RA) is fluent in these two languages but lacks experience speaking in other languages.

### Setting

The study was conducted at four asylum seeker centers in Lithuania, managed by the Ministry of the Interior of the Republic of Lithuania, and the Ministry of Social Security and Labor. Conducting interviews in private spaces within the asylum seeker centers facilitated open and candid discussions. Field notes, capturing participant demographics and key interview points, were taken during interviews and later referenced in reflective conversations between researchers.

### Population

The study targeted undocumented migrants (19 years old or older) who entered Lithuania from the Belarussian border after June 2021, regardless of nationality, gender, or current asylum status. For individual interviews, individuals under 19 and those not proficient in Arabic, or English, were excluded. Nationality and gender were not primary criteria for participant selection to ensure a focus on the shared experiences of healthcare access and social integration challenges among asylum seekers, regardless of their background. This approach aimed to capture a broad range of experiences, reflective of the asylum-seeking population’s challenges in Lithuania.

### Saturation point

The total number of semi-structured interviews aimed for data saturation, ensuring quality responses and conclusive findings. 21 interviews were conducted. Saturation was considered achieved when no new insights emerged from subsequent interviews. Thus, there was no need to conduct more interviews.

### Data analysis

Qualitative data from semi-structured interviews were analyzed thematically using Atlas.ti. In this study, we employed a rigorous thematic analysis approach to identify, analyze, and describe the key themes and subthemes in the data. The transcripts were rigorously cross-checked against the original recordings, as outlined by Braun and Clarke (2006) (31), for the purposes of examining, interpreting, and reporting significant patterns or themes emerging from the data collected (31). One of the researcher team RA meticulously transcribed the audio-recorded interviews verbatim, and the transcripts were rigorously verified for accuracy against their respective source recordings. A qualitative data analysis software called Atlas.ti was utilized to enhance the analysis process (32). RA and RU independently coded the data, assigning codes to salient textual units.

The team participated in a comprehensive discussion, facilitating a consensus-based approach to resolving any discrepancies. This collaborative process ensured the consistency and robustness of the coding process. The codes were then systematically amalgamated into cohesive overarching themes and subthemes, enabling us to explore the participants’ experiences and perspectives. The abduction approach was used throughout the data analysis process, as we iteratively moved back and forth between the data and existing theories, in order to generate new insights and explanations. In accordance with the recommendations of Green et al. (33) and Eakin and Gladstone (34), we generated meaningful insights and advanced our understanding of the research phenomenon by employing a rigorous and systematic approach to data analysis. The researchers played a critical role in the thematic analysis of the data, ensuring rigor and transparency. They independently coded the transcripts, discussed discrepancies, and reached consensus on the themes, which were then cross-checked with the original data. This process ensured a careful examination of the data and the reliability of the findings. Moreover, to mitigate potential biases arising from the researchers’ roles, reflexivity was practiced throughout the study. Researchers (RA, RU) continuously reflected on their assumptions, beliefs, and interactions with participants to ensure these did not unduly influence the data collection, analysis, or interpretation. Peer debriefing and triangulation were also employed to enhance the credibility of the findings.

## Results

The study included 21 participants, encompassing diverse social and demographic characteristics (Table 1). Among the study participants, 13 were men and 8 were women, aged between 19 and 40 years. The marital status of participants varied, with 9 being single and 12 married. Notably, a significant portion of the cohort (12 participants) was accompanied by family members, including siblings, children, spouses. All participants (21) were classified as asylum seekers, with 4 in the process of seeking asylum and 17 currently appealing because of the rejection of their previous applications. The predominant country of origin among the participants was Iraq (18 individuals), followed by Lebanon, India, and Sri Lanka, each represented by 1 participant. The results are presented in two primary thematic areas, each with multiple subthemes that emerged from the analysis of qualitative interviews. These themes encapsulate the complex interplay of healthcare access, mental health, social support, educational opportunities, living conditions, and aspirations amidst the pandemic-induced uncertainty.

**Table 1:** Social and demographic characteristics of the study participants.

	Description	Number of participants
Gender	Men Women	13 8
Marital status	Single Married	9 12
Accompanying family	With family members,i.e. brother, sister, daughter, son, husband,wife	12
Legal status	Asylum seeker Application in process With rejected application/appeal	21 4 17
Country of origin	Iraq Lebanon India Srilanka	18 1 1 1

### Theme I: healthcare needs and the impact of the COVID-19 pandemic

#### Subtheme1: limited access and delays in medical attention

Participants frequently expressed frustrations regarding access to healthcare services. Long waiting times were a common grievance, often exacerbated by harsh conditions. One participant shared,

*“We usually take an appointment with the doctor; however, we have to wait several hours outside in cold weather, people with us may get infected also.”* R5.

Such experiences highlight the difficulties faced in obtaining timely medical attention. The situation is further complicated for those needing specialized care such as dental care taking into consideration the financial barriers, as another participant noted, *“I could not find a dentist in the camp. I have no money for treatment.”* R2.4.

#### Subtheme2: challenges in communication and gender-specific healthcare needs

Communication barriers during medical appointments were shown, with some reliance on fellow asylum seekers for translation:

*“*We have a translator (asylum seeker) who assists us during medical appointments.*”* R10.

*“The real problem is, the doctor does not understand our language, he gives the same pink pill, a painkiller.”* R5.

This *ad-hoc* arrangement raises concerns about the accuracy and confidentiality of medical information. Furthermore, the lack of gender-specific healthcare services was a significant issue, particularly for female asylum seekers, as indicated by the absence of adequate facilities for their specific health needs*: “We need a gynecologist, unfortunately, we do not have one here.” R10.*

#### Subtheme 3: direct impact of COVID-19

##### Testing and isolation protocols

The response to COVID-19 within the camps included regular testing and isolation of positive cases. One participant described the procedure: *“*…*they do COVID-19 testing, if they find a positive result, they move the infected ones into isolation wards.”* R18.

While this indicates some level of proactive measures, the effectiveness and implementation of these protocols remain a concern.

Regarding the COVID-19 related information a participant expressed that the information was printed only in Lithuanian language.

*“We have limited access to information about COVID-19…. all information was in Lithuanian language.”* R9.

##### Vaccination and preventive measures

The approach to COVID-19 vaccination was described as optional, with participants having the choice to be vaccinated: *“Yes, we took the vaccine, they took the names of those who want to be vaccinated, it was optional, not obligatory.”* R13.

However, preventive measures within the camps, especially regarding social distancing, were found lacking. One participant highlighted this concern: *“We panicked, although we received masks and hygiene, there was no social distance.”* R14.

#### Subtheme 4: mental health crisis amidst uncertainty

The mental health impact due to the prolonged pandemic-related uncertainty and restrictive living conditions was significant.

One asylum seeker articulated their distress, saying.

*“I am very stressed and emotionally exhausted, this is my eleventh month in this camp, I am not allowed to get out of it*…” R13 Such statements reflect the psychological turmoil experienced. Mental health concerns on the asylum seekers children *“All refugees have psychological conditions, even my little child, he is so tired, feeling depressed.”* R11.

### Theme II: social needs and aspirations amidst pandemic-induced uncertainty

#### Subtheme 1: impact on asylum application processes

A critical systemic effect of the pandemic where there are delays in assessing the asylum application, as highlighted by participants, was its impact on asylum application procedures. One interviewee succinctly captured this issue: *“The pandemic affected the processing of our asylum applications, causing delays*.*”* R12.

#### Subtheme2: language learning opportunities and challenges

One of the prominent challenges faced by asylum seekers in Lithuania, as revealed by our findings, is the inconsistency and inaccessibility of language education. The struggle to learn the local language in an environment lacking structured educational support is heard in the voices of the participants.

An issue impacting language acquisition for asylum seekers is the lack of consistent scheduling for language classes. Participants expressed frustration with the erratic scheduling, which disrupts the learning process.

One asylum seeker articulated this challenge: *“We want to learn the language, but we do not have a timetable for classes. Every time, they schedule it differently*.*”* R16.

This lack of regularity and predictability in language education further compounds the difficulties faced by asylum seekers in adapting to their new environment.

The mental strain and exhaustion experienced by asylum seekers significantly hinder their ability to engage in language learning effectively. This is poignantly captured by a participant’s statement: *“mastering the language is important for us, however, we do not have a relaxed mindset, we are emotionally exhausted, we have a distributed state of mind, we do not focus on what they say during the class*.*”* R11.

This quote underscores the interplay between mental health and the ability to benefit from educational opportunities.

#### Subtheme 3: challenges in living conditions and basic needs

The quality of food and nutrition emerged as a significant concern among the asylum seekers interviewed for our study. Participants drew attention to several critical issues pertaining to the meals provided in the reception centers. A key aspect highlighted was the lack of variability and cultural sensitivity in the diet offered. In reception centers managed by social services, participants are involved in preparing their own meals, allowing for some degree of personalization. However, in other reception centers, particularly those under the supervision of the army, meals are provided with little consideration for individual dietary preferences or cultural practices. This lack of customization is particularly problematic for individuals from diverse cultural backgrounds who might have specific dietary restrictions, such as not eating certain types of meat or avoiding pork.

*“No halal food, they feel irritated when we ask them for that.”* R6.

*“Indian people do not eat beef, but they serve it here…One social worker tried to change the food for us.”* R16.

The living conditions in the camps, particularly during the pandemic, were described as inadequate and challenging. One participant noted, highlighting the lack of facilities. *“We share kitchens with many families, it is overcrowding…We have many refugees, unfortunately, we do not have enough facilities.”* R14.

The cramped and overcrowding spaces were also a major concern, as another participant described: *“The living conditions here are very bad. I live in a small room with 16 people.”* R16 pointing to the physical constraints, privacy concerns and discomfort experienced.

#### Subtheme 4: restricted freedom and its psychological impact

The restrictions on freedom of movement within the camps had a significant impact on the asylum seekers. The longing for autonomy and better living conditions was palpable in their narratives.

*“We live in an uncertainty, we kept detained for almost six months now, they did not provide any information regarding the detention period…. even if everything will be available here in this camp, we want our freedom back, that’s our only Need”* R14.

*“They treat us as if we are criminals, not refugees. We do not know when we will leave this camp, we do not hold any information to stand for.”* R17.

In spite of the many challenges, the aspirations and hopes of the asylum seekers for a better future remained undiminished. Their narratives reflected a determination to overcome the barriers they faced. The deep desire for a better life was also evident*: “We desperately need people who can consider the situation we are going through every day… we want our freedom back, that’s our only need.”* R10.

## Discussion

The study’s findings-which encompass healthcare access, mental health, language barriers, and living conditions-offer profound insights into the complex realities faced by these individuals. The insights gained from this study not only echo existing research but also uncover new layers of challenges exacerbated by the pandemic. This includes the direct impact of COVID-19, the mental health crisis fuelled by prolonged uncertainty, the struggles with language learning in an already stressful environment, and the systemic effects on asylum application processes. Furthermore, we explored how these challenges aligned with the Social Determinants of Health (SDH) framework, offering a comprehensive understanding of how various socio-economic and political factors shape the health outcomes of undocumented migrants and asylum seekers.

The findings of our study align with the broader landscape of existing research on the health and social needs of undocumented migrants and asylum seekers, particularly focusing on the challenges amplified by the COVID-19 pandemic (1, 35, 36). Undocumented migrants are a vulnerable population group in the context of the COVID-19 pandemic due to increased risk of infection, severe morbidity, and mortality. Studies show that in other countries the pandemic disproportionately affected refugees and asylum seekers, leading to higher rates of COVID-19 transmission and mortality, exacerbated by disparities in healthcare access (1, 37). This underscores the need for policy reforms that prioritize equitable healthcare access, particularly in crisis situations. Our analysis reveals a complex interplay of factors affecting the well-being of this vulnerable group, encompassing healthcare access, mental health, language learning opportunities, and broader social and legal aspects of their lives in Lithuania.

The direct impact of COVID-19 observed in our study correlates with findings from modeling studies (38, 39), which have identified significant challenges in implementing effective testing and isolation protocols in camps housing migrants and refugees. Additionally, these studies note that refugees often face limited access to essential health services and humanitarian aid, including food, water, and shelter (40). An additional concern is the restricted availability of personal protective equipment, such as masks and gloves, in typical refugee settlements (41). This limitation is critical in efforts to prevent the spread of COVID-19 among refugee populations. Our study further underscores these challenges, highlighting the complexity and inadequacy of preventive measures, particularly the scarcity of masks and the absence of social distancing practices. These findings point to a significant gap in the comprehensive implementation of health policies designed to protect asylum seekers during the pandemic. Healthcare providers and social service professionals must receive cultural competency training to effectively address the needs of undocumented migrants and asylum seekers. Understanding the cultural nuances, language preferences, and unique challenges faced by this population is essential for providing equitable and effective care (20, 42).

The significant mental health impact due to prolonged uncertainty and restrictive living conditions reported in our study adds to the growing body of evidence on the mental health challenges faced by migrants (16, 43). Our study contributes to this discourse by linking these mental health challenges interplay with pandemic-related stress, and living conditions thus highlighting the need for targeted mental health interventions that are responsive to the unique circumstances of asylum seekers.

The struggles with language learning and the lack of consistent educational opportunities for asylum seekers, as revealed in our study, offer insights into the overlooked aspect of migrant integration challenges. Our findings indicate a deficit in structured language education, exacerbated by mental stress and unpredictable scheduling. This underscores the necessity for more robust language education programs, tailored to the needs and mental well-being of asylum seekers. Innovative educational practices for language learning among asylum seekers, can include blended learning (44), culturally responsive teaching (45), leveraging technology (46), community-based learning (47), and peer learning (47). These approaches, such as combining online and traditional classroom experiences, adapting content to cultural contexts, and using digital tools for language acquisition, enhance learning effectiveness. However, they necessitate sufficient support in terms of technology access, specialized teacher training, and financial resources to be successfully implemented.

Our study uniquely highlights the systemic impact of the pandemic on the asylum application process, an aspect less explored in previous research. The delays in processing asylum applications, as indicated in our study, reflect broader systemic issues that extend beyond immediate health and social needs, impacting the legal and prospects of asylum seekers. These challenges call for increased investment in asylum systems to address backlogs and ensure fair treatment of asylum seekers (48). The finding is crucial for understanding the full spectrum of challenges faced by this group during the pandemic. Our insights into the living conditions and aspirations of undocumented migrants and reinforce the call for policy changes to improve living standards.

In our study on the healthcare and social needs of undocumented migrants and asylum seekers, we meticulously navigated instances of contradictory data, which revealed the intricate and diverse realities of this group’s experiences. Notably, we observed divergent perspectives on the accessibility of healthcare services; some participants described satisfactory access, while others pointed to substantial barriers. Rather than overlooking these disparities, we engaged in a thorough analysis to uncover the nuanced dynamics at play, enriching our understanding of the participants’ multifaceted experiences.

This deliberate examination allowed us to spotlight critical inconsistencies, particularly in healthcare accessibility and the efficacy of communication with healthcare providers. Our acknowledgment of these contradictions was instrumental in crafting a detailed portrayal of the undocumented migrants and asylum seekers’ lived realities. It facilitated the development of more granular themes, enriching our insights and ensuring our findings faithfully represent the wide spectrum of experiences encountered by our study population.

Furthermore, this meticulous approach underscored our dedication to presenting an authentic and comprehensive analysis of the challenges these individuals face. It highlighted our commitment to contributing substantive, nuanced discourse to the broader conversation on migration and health. Ultimately, by embracing and methodically interpreting contradictory data, we have underscored the importance of designing bespoke interventions that recognize and address the varied needs and experiences of undocumented migrants and asylum seekers, advocating for solutions that are as multifaceted as the challenges they face.

### Social determinants of health (SDH) framework

Our study’s findings can be comprehensively analyzed through the lens of the Social Determinants of Health (SDH) framework, particularly considering the unique experiences of undocumented migrants and asylum seekers. This framework, developed by the Commission on Social Determinants of Health (CSDH) of the World Health Organization, provides a robust model for understanding the complex interplay of various social, economic, and political factors that influence health outcomes.

Our study’s findings reveal how host country policies intricately shape healthcare accessibility for undocumented migrants and asylum seekers. These policies, often not tailored to the unique needs of this populations, result in significant healthcare disparities. This aligns with the SDH framework’s assertion that political systems and socioeconomic factors critically shape health (26). For instance, the lack of policy provisions for language interpretation services in healthcare settings, as highlighted in our study, can be seen as a direct outcome of these structural determinants.

The SDH framework posits gender and racial discrimination as key determinants of health inequities. Our study underlines this by documenting instances where female migrants and those from certain ethnic backgrounds faced additional health challenges. These findings resonate with the SDH emphasis on the role of societal discrimination in shaping health outcomes (26).

The harsh conditions in the undocumented migrant asylum seekers camps, coupled with socio-psychological stressors such as anxiety due to uncertain legal status, reflect the SDH’s intermediary determinants. Our study’s findings on the mental health crises exemplify the impact of these environmental and socio-psychological factors on health outcomes, a correlation strongly emphasized in the SDH framework (26).

The limited access to healthcare services and constraints in making healthy lifestyle choices, as highlighted in our study, fall under the intermediary determinants of health. These factors contribute to unequal exposure to health risks and services, aligning with the SDH framework’s assertion on the role of healthcare systems and behavioral factors in health inequities (26).

Consistent with the SDH framework, our study positions immigration itself as a determinant of health. The undocumented migrants and asylum seekers experiences, marked by challenges such as adaptation to new environments and precarious legal status, interplays with other social determinants, influencing health outcomes. This perspective broadens the scope of SDH, highlighting the need for health policies and practices to consider immigration status as a critical factor in health equity.

## Recommendations

### Healthcare services

It Is important to establish healthcare services that are culturally sensitive and linguistically tailored to the needs of undocumented migrants and asylum seekers. This initiative should include training for healthcare providers in cultural competency and offering language translation services. It’s also imperative to implement healthcare services that address the unique needs of female asylum seekers, including reproductive health and support for survivors of gender-based violence. Additionally, to create dedicated services to cope with mental health problems and cater to the psychological stresses experienced by this group. Moreover, enhancing healthcare access for undocumented migrants and asylum seekers by reducing waiting times, offering mobile health clinics, and providing specialized medical services is crucial. Establish outreach programs to educate undocumented migrants and asylum seekers about their rights to healthcare services and how to access them. Use community leaders and organizations to facilitate trust-building and communication.

### Social services and integration

It is crucial to provide consistent and structured language education programs to facilitate the integration of asylum seekers and migrants into society. Address living conditions in asylum centers by improving aspects such as reducing overcrowding, enhancing the quality of nutrition, and respecting cultural dietary preferences. Create and maintain social support networks that provide emotional support, legal advice, and assistance with navigating healthcare and social services. Ensuring the ability to practice cultural and religious traditions helps maintain a connection to their heritage and supports psychological wellbeing. Spaces where cultural and religious practices can be freely observed are important for community cohesion. Conduct workshops and seminars to raise awareness about the cultural backgrounds of undocumented migrants and asylum seekers among the local population to foster a more inclusive community. Advocating for the rights and needs of undocumented migrants and asylum seekers at local and national levels. The ability to work legally and under fair conditions is a critical need. Initiatives to provide vocational training and job placement services can help undocumented migrants gain employment that offers dignity and a living wage.Use media campaigns to raise awareness of their challenges. Furthermore, policies restricting the freedom of movement within asylum centers should be reviewed and revised to promote better psychological health and autonomy.

### Policy and advocacy

Streamlining the asylum application process is essential, ensuring efficiency and transparency, especially considering the challenges posed by the COVID-19 pandemic. Advocating for inclusive health policies that recognize and address the unique challenges faced by undocumented migrants and asylum seekers is necessary. It is recommended to engage migrant communities in decision-making processes to ensure their needs and voices are represented and empower them through community leadership programs. Foster partnerships between governmental bodies and NGOs to create integrated support systems for migrants. Additionally, collaboration with NGOs and international organizations is vital to provide comprehensive support. Increase funding for public health services and NGOs working with undocumented migrants and asylum seekers to ensure they have the necessary resources to meet their healthcare and social needs. Continuous research and monitoring of the evolving health and social needs of undocumented migrants and asylum seekers will ensure the effectiveness and relevance of services and policies. Public awareness campaigns should be conducted to increase understanding and empathy for the challenges faced by migrants and asylum seekers. Offering legal assistance to asylum seekers and undocumented migrants, along with advocating for their rights and equitable treatment, is also fundamental.

### Strengths and limitations

#### Strengths of the study

Our study employed the Social Determinants of Health framework, offering a comprehensive lens through which to view the health inequities experienced by migrants and asylum seekers in Lithuania. This approach allowed for a nuanced understanding of the complex interplay of social, economic, and political factors influencing health outcomes in this vulnerable population.

The focus on undocumented migrants and asylum seekers, particularly during the COVID-19 pandemic, provided valuable insights into the experiences and needs of a group often overlooked in health research. The qualitative methodology, involving semi-structured interviews conducted in languages familiar to the participants, ensured a depth of understanding and cultural sensitivity in data collection.

The use of Atlas.ti for thematic analysis underscored the methodological rigor of our study, enabling a systematic and detailed examination of the interview data. The diversity in participant demographics enhanced the richness and applicability of our findings within the context of Lithuania.

#### Limitations of the study

Despite these strengths, our study has limitations. The specific focus on Lithuania limits the generalizability of our findings to other contexts or migrant populations. An additional limitation is that our study only included participants proficient in Arabic or English, excluding other asylum seekers who might have provided diverse perspectives but were unable to participate due to language constraints. Furthermore, our study focused exclusively on adult individuals, thus excluding children. This exclusion means that the experiences and needs of a significant segment of the asylum-seeking population in Lithuania, particularly those of children who often face unique challenges, were not explored.

While this study offers important insights into the healthcare and social needs of undocumented migrants and asylum seekers in Lithuania, particularly in the context of the COVID-19 pandemic, the findings should be interpreted with these limitations in mind. The strengths of the study lie in its comprehensive approach and focus on a vulnerable population, while its limitations point to areas for further research and methodological refinement, including the experiences of children and non-Arabic or English-speaking migrants.

#### Conclusion

In conclusion, our study contributes to the understanding of the challenges faced by undocumented migrants and asylum seekers in Lithuania, particularly in the context of a global pandemic. It highlights the urgent need for comprehensive policies and practices that are grounded in the principles of equity, compassion, and human rights. The intermediary determinants, such as living conditions, psychological stressors, and healthcare system factors, have further exacerbated their vulnerabilities, as evidenced by the mental health challenges and restricted access to essential services.

Healthcare services must be culturally sensitive and linguistically accessible, catering to the specific needs of diverse groups-including women and children. Additionally, social services, including language education and assistance with integration into the host society, are crucial for fostering a sense of belonging and community.

## Data availability statement

The original contributions presented in the study are included in the article/Supplementary material, further inquiries can be directed to the corresponding author.

## Ethics statement

The studies involving humans were approved by the Ethical committee of the Department of Nursing of the Institute of Health Sciences of the Faculty of Medicine of Vilnius University (150000-KP-47). The studies were conducted in accordance with the local legislation and institutional requirements (49). The participants provided their written informed consent to participate in this study.

## Author contributions

RA: Conceptualization, Data curation, Formal analysis, Investigation, Methodology, Supervision, Writing – original draft, Writing – review & editing. RU: Formal analysis, Investigation, Methodology, Project administration, Software, Writing – review & editing. AJ-A: Formal analysis, Investigation, Methodology, Writing – review & editing. MS: Formal analysis, Investigation, Methodology, Writing – review & editing. DA: Formal analysis, Investigation, Methodology, Writing – review & editing. EB-V: Formal analysis, Investigation, Methodology, Writing – review & editing. ER-A: Formal analysis, Investigation, Methodology, Supervision, Writing – review & editing. NI: Conceptualization, Formal analysis, Investigation, Methodology, Supervision, Writing – review & editing.

## Appendix 1: Glossary on Migration

**Table:**

Term	Meaning
Asylum seeker	An individual who is seeking international protection. In countries with individualized procedures, an asylum seeker is someone whose claim has not yet been finally decided on by the country in which he or she has submitted it. Not every asylum seeker will ultimately be recognized as a refugee, but every recognized refugee is initially an asylum seeker.
Internally displaced persons	Persons or groups of persons who have been forced or obliged to flee or to leave their homes or places of habitual residence, in particular as a result of or in order to avoid the effects of armed conflict, situations of generalized violence, violations of human rights or natural or human-made disasters, and who have not crossed an internationally recognized State border.
Migrant	At the international level, no universally accepted definition for “migrant” exists. The term migrant was usually understood to cover all cases where the decision to migrate was taken freely by the individual concerned for reasons of “personal convenience” and without intervention of an external compelling factor; it therefore applied to persons, and family members, moving to another country or region to better their material or social conditions and improve the prospect for themselves or their family. The United Nations defines migrant as an individual who has resided in a foreign country for more than one year irrespective of the causes, voluntary or involuntary, and the means, regular or irregular, used to migrate. Under such a definition, those travelling for shorter periods as tourists and businesspersons would not be considered migrants. However, common usage includes certain kinds of shorter-term migrants, such as seasonal farmworkers who travel for short periods to work planting or harvesting farm products.
Refugee	A person who, “owing to a well-founded fear of persecution for reasons of race, religion, nationality, membership of a particular social group or political opinions, is outside the country of his nationality and is unable or, owing to such fear, is unwilling to avail himself of the protection of that country.
Undocumented migrant	A non-national who enters or stays in a country without the appropriate documentation. This includes, among others: a person (a) who has no legal documentation to enter a country but manages to enter clandestinely, (b) who enters or stays using fraudulent documentation, (c) who, after entering using legal documentation, has stayed beyond the time authorized or otherwise violated the terms of entry and remained without authorization.
